# Authoritarian leadership and nurse presenteeism: the role of workload and leader identification

**DOI:** 10.1186/s12912-022-01119-2

**Published:** 2022-12-02

**Authors:** Geyan Shan, Wei Wang, Shengnan Wang, Yongjun Zhang, Shujie Guo, Yongxin Li

**Affiliations:** 1 Business School, Henan University, Kaifeng, China; 2 Institute of Psychology and Behavior, Henan University, Kaifeng, China; 3 Department of Outpatient, Henan Provincial People’s Hospital, People’s Hospital of Zhengzhou University, Zhengzhou, China; 4 Institute of International Education, Henan University of Animal Husbandry and Economy, Zhengzhou, China

**Keywords:** Presenteeism, Authoritarian leadership, Leader identification, Workload, Nurse

## Abstract

**Background:**

Nurses’ health in the workplace is crucial for ensuring the quality of healthcare. However, presenteeism, the behavior of working in a state of ill health, is widespread in the nursing industry. Considering that the origin of authoritarian leadership and the prevalence of presenteeism are inseparable from Chinese workplace culture, this study aimed to explore the impact and mechanism of authoritarian leadership on presenteeism.

**Methods:**

A total of 528 nurses were recruited from four grade III level A hospitals in the present survey, which was distributed across 98 nursing teams. Participants were required to complete self-report measures on authoritarian leadership, presenteeism, workload, and leader identification. Description, correlation, and multilevel linear regressions were applied for data analysis.

**Results:**

The present study found that presenteeism was significantly related to participants’ demographic characteristics, such as marital status, educational level, technological title, and general health. There was a positive relationship between authoritarian leadership and presenteeism, and workload acted as a mediator in authoritarian leadership and presenteeism. Furthermore, leader identification moderated the relationship between authoritarian leadership and workload. When nurses were under high leader identification, the positive impact of authoritarian leadership on workload was reinforced.

**Conclusions:**

This study revealed the potential antecedents and mechanisms of nurse presenteeism from the perspective of workplace culture. Results indicated that the excessive authoritarianism of leaders and the heavy workload faced by nurses may be the significant triggers for nurses’ presenteeism. The role of leader identification is not always protective, which may heighten the relationship between dark leadership and its outcomes. These observations contribute to enriching research on presenteeism and authoritarian leadership, and provide valuable insights for cultivating healthy working behaviors.

## Background

Health is a fundamental right of every human being, while also being an inevitable requirement for promoting an individual’s overall development. Employees constitute one of the most important resources of organizations [1]; thus, maintaining employees’ health is crucial for the sustainable development of organizations. However, presenteeism, the behavior of working in a state of ill health [2], has become a widespread phenomenon in the workplace, which has attracted the attention of multidisciplinary researchers in the fields of industrial and organizational psychology, occupational health psychology, epidemiology, and nursing management in recent years [3]. Presenteeism has been defined as the behavior of people who still turn up at their jobs despite complaints of ill health that should prompt rest and absence from work [4]. Its prevalence has been documented in more than a dozen countries, such as the US, Canada, the UK, the Netherlands, Spain, and China, with presenteeism rates ranging from 30% to more than 90% [5]. Existing studies have shown that nurses tend to experience a high incidence of presenteeism [4, 6], despite being equipped with abundant health knowledge and high levels of health literacy [7]. For example, the occurrence of presenteeism among Dutch nurses reached 50% [8], and the overall presenteeism rate was 85.5% among nursing students in the USA, Japan, and South Korea [9]. Whereas the incidence of presenteeism among Chinese nurses reached 94.25% [7]. Furthermore, nurses working under unhealthy conditions (i.e., presenteeism) tends to lead to a series of negative consequences for the health and productivity of individuals, safety of their patients, and development of organizations. For instance, it may affect the healthy recovery of nurses [10], increase the number of falls in patients and drug errors [11], and cause financial burden and productivity loss in medical organizations [7].

Considering the severely negative outcomes that nurses’ presenteeism can cause in multiple fields, it is essential to explore its occurrence mechanism. Previous research has preliminarily examined leader-related factors that are closely related to presenteeism, such as leader behavior, leader pressures, and leader–follower relationships [12–14]. Moreover, the impact of leadership on subordinates’ behaviors is also noticeable in that leadership would likely play a vital role in shaping the healthy work behaviors of subordinates. Limited empirical research showed that health-promoting leadership and supportive leadership behavior were conducive for reducing the incidence of employees’ presenteeism [15, 16]. From the theory of paternalistic leadership [17], the work team is analogous to a family in which a leader acts as the father with two typical characteristics, majesty and mercy, while the subordinates play the role of a child, thereby reflecting the concept of “superior and inferior” in traditional Chinese culture. To some extent, supportive leadership behaviors and health-promoting leadership are more similar to the merciful father side of leaders, which provides guidance and supports work resources for subordinates. However, nursing management is also characterized by a strict rank and authority that embodies the majestic father side of leaders, and its role mechanism on presenteeism has not been extensively examined. To understand the entire picture of the influence of leaders on subordinates in the Chinese cultural context, this study mainly examined the impact mechanism of authoritarian leadership on nurses’ presenteeism. Authoritarian leadership refers to a leadership style that emphasizes the use of authority to control one’s subordinates [18], which requires employees to obey and follow the leader’s teachings to ensure efficient operation of the organization [19]. Hence, the authoritarian leadership prevalent in the nursing field may encourage nurses to prioritize their career over their health, leading to presenteeism.

Existing studies have demonstrated that leaders’ particular behaviors or leadership style can inherently be either stressful or positive for subordinates, and can consequently influence their levels of stress and affective wellbeing [20]. According to the main features of authoritarian leadership, authoritarian leaders expect unquestioning obedience, thereby controlling information and restricting subordinates’ autonomy [21], which can lead to more job demands for subordinates. Moreover, previous research has demonstrated that a heavy workload is a crucial factor in the occurrence of presenteeism [8, 22]. Thus, authoritarian leadership may have an indirect impact on presenteeism through increasing employees’ workload. Meanwhile, identification with leaders implies that the employees consider the leader as a self-reference point or model of self-definition, and have acceptance of the leader’s perception and attitude [23]. When subordinates strongly identify with their leader, they would respect them, feel proud of them, and will be more likely to exhibit behaviors that are encouraged by the leader [24]. Therefore, when nurses have high identification with their leader, they may accept more organizational tasks and a heavier workload, as expected by the authoritarian leader. In contrast, when subordinates rarely identify with leaders, despite their workload being affected by authoritarian leaders, they hardly take the initiative to undertake additional tasks according to authoritarian leaders’ expectations. As a consequence, the correlation between authoritarian leadership and workload would be stronger under high leader identification rather than low, and leader identification may moderate the relationship between authoritarian leadership and workload. In summary, the present study aims to explore the occurrence mechanism of presenteeism and focused on the impact of authoritarian leadership in the Chinese workplace culture, which would contribute to examining the impact of a leader’s authoritative side on presenteeism and enrich the application of paternalistic leadership theory. Furthermore, this study draws an overview of the relational mechanism of authoritarian leadership and presenteeism through the combination of the paternalistic leadership theory and conservation of resources theory, which would be instructive in the effective implementation of nursing management to prevent and reduce nurse presenteeism.

### Authoritarian leadership and nurse’s presenteeism

Presenteeism is more prevalent among nurses, compared with other occupational groups; this could be attributed to the characteristics of nursing work, such as high stress, night shift work, and low substitutability [4, 25, 26]. Previous studies have indicated that the prevalence of presenteeism reached 94.25% among Chinese nurses [7]. Furthermore, multiple negative consequences result from presenteeism among nurses who undertake vital tasks in the healthy development of nationals. For example, nurse presenteeism could impair their health and well-being [27], pose a high risk to their patients and work environment [25], and even cause productivity and economic losses to organizations and society [7, 28]. Therefore, paying attention to nurses’ presenteeism and its causes would be conducive to promoting individuals’ health and the quality of healthcare services.

In the work-related value system embedded in the Confucian tradition of China, although some effort-related work values (e.g., endurance, persistence, and hard work) may enhance work outcomes among employees, they could also contribute to a “long-hour working culture” and the high prevalence of presenteeism [29]. Moreover, the Chinese culture also attaches importance to hierarchy; therefore, the relationship between leaders and subordinates follows a superior/inferior rationale, wherein leaders control the resources and fate of subordinates [30], which nourishes authoritarian leadership. Based on the theory of paternalistic leadership, the typical characteristics of authoritarian leaders could be considered as comprising four aspects: the autocratic style that manifests as grabbing power, controlling information, and strictly monitoring subordinates; derogate the ability of subordinates that manifests as willful disregard for subordinates’ contributions and suggestions; image decoration that manifests as manipulating information to create a good image; and instructional behaviors that manifest as emphasis on the importance of performance and providing guidance to ensure subordinates high performance [18]. Existing research indicates that the effect of authoritarian leadership on organizations and subordinates is controversial. On one side, an authoritarian leader is dedicated to ensuring the efficient operation of an organization, demonstrates high performance standards for subordinates, and promotes subordinates to agree with and complete assignments [18, 31], which may result in rapid completion of tasks, performing work accurately, and meeting the organizational performance standards in a timely manner. On the flip side, an authoritarian leader is canonical and unchallenged while strictly controlling their subordinates and berating dissent [18]. From the perspective of subordinates, the strict requirements and tight monitoring from authoritarian leaders tend to trigger feelings of uncertainty and decrease the subordinates’ psychological safety [32, 33]. As a result, when employees feel sick, to relieve the sense of insecurity, they are inclined to resort to presenteeism [26]. Combined with the conservation of resources theory [34], the underlying threats of psychological resource loss that evocated from the strict requirements and tight monitoring of authoritarian leaders would increase pressure and tension for subordinates. When individuals are in poor health, presenteeism would be considered an effective way to maintain the existing resources and confront the psychological threat from strict controls and intensive surveillance. Moreover, authoritarian leaders signal a strong disregard for the interests and perspectives of their subordinates [35, 36], consequently neglecting the health complaints of subordinates and encourage those with poor health to guard collective benefits, thereby generating more presenteeism behaviors of subordinates. Consequently, we proposed the following hypothesis:

- *Hypothesis 1: Authoritarian leadership would be positively associated with nurses’ presenteeism.*

### Mediation effect of workload

According to the conservation of resources theory [37], people strive to retain, protect, and build resources, and psychological stress occurs with the potential or actual loss of these valued resources. Resources refer to those objects, conditions, personal characteristics, and energies that are valued by the individual or that serve as a means for the attainment of these objects, conditions, personal characteristics, or energies. Workload, a stressor in a work environment, consumes psychological, physical, or other valued personal resources. It represents a demand pressed on employees that is only met through the continual consumption of resources [38], which reflects the work demands that individuals perceive as being placed upon them [39]. From this perspective, when an individual is in poor health, presenteeism can allow them to maintain the existing resource level to cope with the loss of job-related resources. Continuing to work when sick would be an effective way to capitalize on other available resources. In other words, since heavy workloads have to be met in order to perform adequately, employees will be inclined to do everything they can to meet these demands so that their performance remains at the desired level [8]. Existing empirical research has also demonstrated that workloads exhibited strong positive correlations with presenteeism, whether in the general perceived workload or the quantitative demands placed on individual [8, 22, 40]. Therefore, the potential resource threatens that heavy workload brought is likely to be a vital trigger of presenteeism.

Excessive demands imposed by the organization or leader are likely to result in work overload for subordinates [41]. As one of three elements in paternalistic leadership, a typical leadership style in Chinese societies, authoritarian leadership emphasizes leaders’ awe-inspiring behaviors, including powerfully subduing their subordinates, authority and control, intention hiding, rigorousness, and doctrine [18]. On one hand, the characteristics of strong pressure and high control among authoritarian leaders may increase employees’ job pressure and decrease their resources [42]. Following the conservation of resources theory, such stressed leadership tends to trigger resource threats as well as individual stress responses such as burnout, which may increase individuals’ sense of overload. In contrast, the theory of paternalistic leadership indicates that authoritarian leaders emphasize their authoritative position and power to perform tasks regardless of subordinates’ conditions [32]; thus, authoritarian leaders are oriented toward work results and tend to express elevated job demands to their subordinates. Consequently, authoritarian leadership may increase nurses’ workloads, which may further facilitate the prevalence of presenteeism. The following second hypothesis was proposed:

- *Hypothesis 2: Authoritarian leadership would increase nurses’ presenteeism via aggravating their workload.*

### Moderation effect of leader identification

Leader identification concerns a person’s perception of “oneness” with the leader [43], which reflects the extent to which a follower’s beliefs about the leader are self-defining or self-referential [44, 45]. To a certain degree, the identification of leaders plays a role in the effectiveness of leadership [46]. Since the leader is the spokesperson of their organization, the identification of leaders leads to employees being more willing to abide by the norms and values of the organization [47]. Subordinates with high leader identification are likely to accept leaders’ goals as their own and conform to their will [48]. Meanwhile, when subordinates identify with their leader, they tend to align their interests with those of the leader and produce a strong desire to contribute to the leader’s goals and success [49]. When nurses have high identification with their leader, they may shoulder more organizational tasks and job demands on their own initiative to meet the expectations and interests of highly authoritarian leaders. The more work stress and tasks they seek to undertake under such conditions, the heavier workload they would perceive invisibly. On the contrary, the workload of nurses who have weak identification with their leader may be less susceptible to the will of authoritarian leaders, due to the bottom level of initiative for contributing to the leader’s success. Accordingly, leader identification would play a moderating role between authoritarian leadership and workload, and the third hypothesis was proposed as follows:

- *Hypothesis 3: Leader identification would moderate the relationship between authoritarian leadership and workload, and for the nurses with high level of leader identification, the relationship would be strengthened.*

**Fig. 1:**
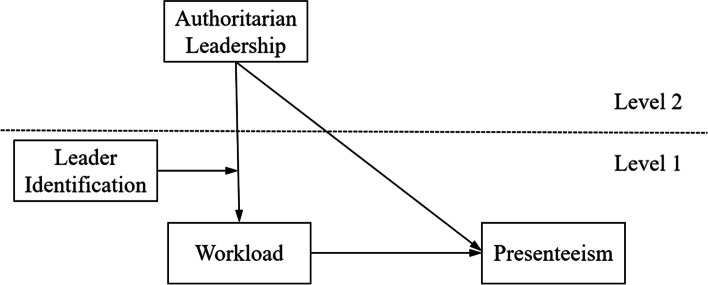
Hypothesized conceptual model

As outlined above, to reveal the relationship between the localization leadership, in the Chinese cultural context, and nurses’ presenteeism, the present study was designed to explore the direct cross-level influence of authoritarian leadership on subordinates’ presenteeism. Simultaneously, the indirect impact of authoritarian leadership on nurses’ presenteeism was also examined, specifically regarding the mediated effect of workload and the moderated effect of leader identification. The integrated conceptual model is illustrated in Fig. 1.

## Methods

### Procedure and participants

In the present study, paper questionnaires were distributed to 660 nurses of 110 nursing teams from four large grade III level A hospitals located in Henan Province, China, using convenience sampling methods. The grade III level A hospital refers to a medical and prevention technology center with comprehensive medical, teaching, and scientific research capabilities, representing advanced medical level and aiming to provide the highest level of medical and health services in the region. The investigation was conducted from September to December of 2020. Prior to the investigation, the research group selected target hospitals and contacted related nursing management departments to introduce the research purpose and plan. After obtaining permission from the hospital’s nursing management, two investigation teams were formed comprising two researchers in each team who are graduate students of psychology and have been uniformly trained. Then researchers arrived at each department, explained instructions for completing the questionnaires, and distributed the paper questionnaires, which had been bound and coded in advance, to the participants, with the guidance and coordination of nurses in the sampling hospital. Finally, the completed questionnaires were returned to the researchers by the participants, following which a simple check to ensure the quality of data was performed. All participants provided informed consent before completing the questionnaire.

After data arrangement and data cleaning, 528 valid responses from 98 nursing teams remained, and the effective response rate was 80.00%. Among these nurses, 516 (97.73%) were female, 316 (59.85%) were married, and 404 (76.51%) had a bachelor’s degree or higher. The average age of the nurses was 30.16 years (*SD* = 4.83), and their average tenure in nursing was 8.31 years (*SD* = 5.23). Regarding technical titles, 97 (16.29%) had the title of nurse, 196 (37.12%) had the title of nurse practitioner, and 230 (43.56%) had the title of nurse-in-charge or above. The department where they worked covered internal medicine, surgery, pediatrics, obstetrics and gynecology, emergency, and others.

### Measures

General demographic data, such as gender, age, tenure, marital status, educational level, technological title, and general health, were collected. Age and tenure were directly filled in the questionnaire and analyzed as continuous variables. Other information was collected in the form of multiple choices. Gender was divided into male and female; marital status fell into unmarried and married. The educational level was divided into three categories—college and below, bachelor, and master and above. The technical title was divided into three categories, namely nurse and below, nurse practitioner, and nurse-in-charge and above. General health was measured with one item (In general, how would you say your health has been in the past six months?) on a three-point response scale ranging from 1 (“good”) to 3 (“bad”).

Authoritarian leadership was measured using the Authoritarian Leadership Scale, which is the central subscale of the Paternalistic Leadership Scale developed by Cheng et al. [42]. The scale contains eight items rated on a seven-point Likert scale ranging from 1 (“strongly disagree”) to 7 (“strongly agree”). A sample item is “Our team leader (chief nurse) decided all matters individually.” In this study, the Cronbach’s α for this scale was 0.89 and the McDonald’s ω was 0.89.

Leader identification was assessed using the Leader Identification Questionnaire developed by Shamir et al. [50], which contained seven items rated on a five-point Likert scale ranging from 1 (“strongly disagree”) to 5 (“strongly agree”). A sample item is “I trust my leader’s (chief nurse) judgment and decisions completely.” This questionnaire has been widely adopted in the Chinese context and has exhibited good reliability and validity. In this study, the Cronbach’s α for this scale was 0.96 and the McDonald’s ω coefficient was 0.97.

Nurses’ workload was evaluated using the Role Overload Scale developed by Peterson et al. [51], which contains five items rated on a five-point Likert scale ranging from 1 (“strongly disagree”) to 5 (“strongly agree”). A sample item is “It will be necessary to reduce some of my work duties.” The scale has been widely adopted in the Chinese context to measure workload and has exhibited good reliability and validity [52]. In this study, the Cronbach’s α for this scale was 0.92 and the McDonald’s ω coefficient was 0.92.

Presenteeism was surveyed using the Nurse Presenteeism Questionnaire (NPQ) [53], which contains 11 items and aims to measure the occurrence of presenteeism among nurses. An example item is, “Although you had a fever, you still persevered in going to work.” Participants were required to evaluate the frequency with which they had experienced presenteeism during the past half a year, and each item was rated on a four-point scale (0 = “never,” 1 = “once,” 2 = “2–5 times,” 3 = “more than 5 times”), with high scores describing more frequent instances of presenteeism. In this study, the Cronbach’s α for this scale was 0.94 and the McDonald’s ω coefficient was 0.94.

### Reliability and validity analysis

The measurement items in this study are from maturity scales and have considerable content validity. In our study, both the Cronbach’s α and McDonald’s ω coefficients for all scales exceed 0.9, demonstrating excellent internal consistency reliability. Furthermore, confirmatory factor analysis was performed separately for each scale after passing the KMO and Bartlett’s test of sphericity. The results showed favorable construct validity and convergent validity indicating that all items fall on the corresponding factor, and the standardized factor loading, average variance extracted (AVE), and composite reliability (CR) were acceptable. Table 1 shows the results of the reliability test and confirmatory factor analysis for each scale used in this study. Additionally, confirmatory factor analysis was conducted for all items to test the discriminant validity of concepts. Results showed that the four-factors model has a good fit (χ2/*df* = 2.67, CFI = 0.95, TLI = 0.94, GFI = 0.87, NFI = 0.92, IFI = 0.95, RMSEA = 0.06, SRMR = 0.041), which indicates good discriminant validity of the scales.

**Table 1:** The results of the reliability and validity analysis

Variables	Items	Standardized Factor Loading	Cronbach’s α	McDonald’s ω	AVE	CR
Authoritarian leadership	AL1	0.78	0.89	0.89	0.61	0.92
	AL2	0.54				
	AL3	0.92				
	AL4	0.83				
	AL5	0.88				
	AL6	0.89				
	AL7	0.46				
	AL8	0.82				
Leader identification	LI1	0.89	0.96	0.97	0.80	0.96
	LI2	0.78				
	LI3	0.94				
	LI4	0.94				
	LI5	0.93				
	LI6	0.89				
	LI7	0.91				
Workload	WL1	0.78	0.92	0.92	0.71	0.92
	WL2	0.77				
	WL3	0.89				
	WL4	0.92				
	WL5	0.83				
Presenteeism	NPQ1	0.80	0.94	0.94	0.58	0.94
	NPQ2	0.73				
	NPQ3	0.74				
	NPQ4	0.76				
	NPQ5	0.69				
	NPQ6	0.81				
	NPQ7	0.84				
	NPQ8	0.76				
	NPQ9	0.75				
	NPQ10	0.72				
	NPQ11	0.81				

### Statistical analysis

The software G*power version 3.1 was used to calculate the minimum sample size required for the hypothesized model. The effect size f^2^ was set at 0.15, the significance level (*α*) was set at 0.05, the power was set at 0.95, and the number of total predictors was set at 3. Results showed that 119 samples were required to validate the hypotheses of this study, which indicates that the 528 samples included in our study were completely adequate for statistical analysis. The Statistical Package for the Social Sciences (SPSS version 22.0), Mplus, and HAD software were used for data sorting and analysis [54]. Specifically, descriptive statistics were applied to analyze the demographic and research variables. Chi-square or *t-*tests were used to evaluate presenteeism against the demographic variables. Subsequently, combined with the content of this study, the applicability of the data was assessed, such as the intraclass correlation coefficient (ICC) and the within-group agreement (r_WG_) of between-group variables. Finally, the correlation among the variables was evaluated, and a multilevel linear regression was conducted to verify the hypothesized model of this study.

## Results

### Applicability of research data

Considering that all questionnaires in this study were completed by nurses, common method bias was analyzed by controlling for the effects of an unmeasured latent method factor [55]. A latent method factor was constructed based on the original four-factors structure, and all items were allowed to be loaded on it. The latent factor did not correlate with the other factors. The variance explained by the latent method factor was 6.76%, which was lower than the 25% median score reported in previous studies [56]. Therefore, serious common method biases were not observed in this study.

Although the questionnaires were filled in by nurses, authoritarian leadership was a variable at the team level, and data aggregation was required before the regression analysis. We evaluated within-team consistency before aggregating authoritarian leadership at the between-team level. The results showed that the mean within-group agreement r_WG_ for authoritarian leadership was 0.89, ICC (1) was 0.22, and ICC (2) was 0.60. If r_WG_ > 0.70, ICC (1) > 0.12, and ICC (2) > 0.50, significant differences exist in the between-group variances, and these variables can be aggregated [57]. In addition, the ICC (1) of nurse workload and presenteeism were 0.28 and 0.27, respectively. Thus, the hierarchical linear model is suitable for examining the impact of authoritarian leadership on workload and presenteeism across groups.

### Nurses’ presenteeism and differences in demographic characteristics

The overall mean score of the NPQ was 1.42 ± 0.85. Table 2 presents the descriptive results and differences in NPQ scores according to nurses’ demographic characteristics. As shown in Table 1, nurses with different marital status exhibited significant variance in NPQ scores; more married nurses preferred to work while sick, compared with unmarried nurses (*t* = -2.26, *p* < 0.05). Moreover, nurses of different educational level (*F* = 3.46, *p* < 0.05), technological title (*F* = 6.73, *p* < 0.01), and general health (*F* = 33.10, *p* < 0.001) had significant differences in NPQ scores. Further post-hoc analysis indicated that the presenteeism of nurses with a bachelor’s degree was significantly higher than that of nurses with lower educational levels. The higher their title and the worse their health status, the higher the prevalence of presenteeism among the nurses.

**Table 2:** Differences of NPQ scores in demographic characteristics

Variables	Categories	Cases	*M* ± *SD*	*t/ F*	*p*
Gender	Male	12	1.17 ± 0.83	-1.00	0.318
	Female	516	1.42 ± 0.85		
Marital status	Unmarried	175	1.29 ± 0.84	-2.26	0.024
	Married	316	1.48 ± 0.84		
Educational level	College and below	48	1.12 ± 0.68	-2.87	0.005
	Bachelor and above	404	1.43 ± 0.87		
Technological title	Nurse and below	97	1.17 ± 0.82	6.73	0.001
	Nurse Practitioner	196	1.40 ± 0.81		
	Nurse-in-charge and above	230	1.54 ± 0.88		
General health	Good	279	1.15 ± 0.80	33.10	< 0.001
	Middle	183	1.65 ± 0.82		
	Bad	44	1.95 ± 0.70		

### Correlation analysis of research variables

Table 3 presents a correlation matrix for each research variable. As shown in Table 2, leader identification was negatively correlated with workload (*r* = -0.23, *p* < 0.01) and presenteeism (*r* = -0.13, *p* < 0.01), while workload was positively correlated with presenteeism (*r* = 0.47, *p* < 0.01).

**Table 3:** Correlations among research variables

Variables	*M* ± *SD*	1	2	3	4	5	6	7	8	9	10
**Level 1**											
1. Age	30.16 ± 4.83										
2. Tenure	8.31 ± 5.23	0.94**									
3. Gender	1.98 ± 0.15	0.10*	0.09*								
4. Marital status	1.64 ± 0.48	0.63**	0.59**	0.05							
5. Educational level	1.91 ± 0.33	0.17**	0.08	0.09	0.23**						
6. Technical title	2.25 ± 0.75	0.69**	0.66**	0.10*	0.58**	0.20**					
7. General health	1.54 ± 0.65	0.12*	0.11*	0.02	0.01	0.07	0.14**				
8. Leader identification	4.49 ± 0.63	-0.02	-0.01	0.01	-0.02	0.01	0.03	-0.08			
9. Nurse workload	2.86 ± 0.86	0.14**	0.12**	-0.02	0.12*	0.16**	0.10*	0.32**	-0.23**		
10. Nurse presenteeism	1.42 ± 0.85	0.14**	0.13**	0.04	0.10*	0.12**	0.16**	0.34**	-0.13**	0.47**	
**Level 2**											
11. Authoritarian leadership	3.08 ± 0.76	0.18	0.13	-0.22	0.25	-0.07	0.21	0.35*	-0.57**	0.50**	0.39**

*N* = 528, nurses nested in 98 teams; Age and tenure were continuous variables; Gender: 1 = male, 2 = female; Marital status: 1 = unmarried, 2 = married; Educational level: 1 = college and below, 2 = bachelor, 3 = master and above; Technical title: 1 = nurse and below, 2 = nurse practitioner, 3 = nurse-in-charge and above; General health: 1 = good, 2 = middle 3 = bad; *p < 0.05, **p < 0.01, ***p < 0.001

### Hypothesis testing

Table 4 shows the results of the hierarchical linear model. Considering the significant correlation between demographic characteristics and presenteeism, we controlled for these demographic variables in the models. Furthermore, correlation analysis showed that tenure was highly correlated with age, marital status, and technical title (*r* > 0.6). Hence, to avoid high collinearity in these demographic variables, we used tenure, educational level, and general health as control variables in the subsequent models.

**Table 4:** Results of the hierarchical linear model

Variables	Nurse Workload			Nurse Presenteeism		
	**Model 1**	**Model 2**	**Model 3**	**Model 4**	**Model 5**	**Model 6**
Tenure	0.02(0.06)*	0.01(0.01)*	0.01(0.01)	0.02(0.01)*	0.02(0.01)*	0.01(0.01)
Education Level	0.26(0.11)**	0.25(0.11)*	0.24(0.11)*	0.25(0.10)**	0.25(0.10)*	0.18(0.10)
General Health	0.35(0.06)***	0.35(0.06)***	0.35(0.06)***	0.26(0.06)***	0.26(0.06)***	0.17(0.06)**
Authoritarian Leadership		0.29(0.07)***	0.30(0.07)***		0.24(0.06)***	0.25(0.06)***
Nurse Workload						0.28(0.06)***
Leader Identification			-0.33(0.09)***			
AL × LI			0.25(0.07)**			

The numeric in parentheses are standard errors (*SE*) of regression coefficients (*γ*); *AL *Authoritarian leadership, *LI *Leader identification; *p < 0.05, **p < 0.01, ***p < 0.001

As shown in Table 4, authoritarian leadership could influence presenteeism across levels (Model 5: *γ* = 0.24, *p* < 0.001). Therefore, Hypothesis 1 was supported. The results of the hierarchical linear model indicated that authoritarian leadership had a positive impact on nurses’ workload (Model 2: *γ* = 0.25, *p* < 0.001), whereas workload had a positive impact on presenteeism (Model 6: *γ* = 0.28, *p* < 0.001), which rudimentarily supported the mediation effect of workload. Furthermore, the cross-level mediation-lower-level mediator model was established, which adopted multilevel structural equation model in the software of Mplus, and the product-of-coefficients method was used to evaluate the mediation effect of workload on the relationship between authoritarian leadership and presenteeism. Results showed that the indirect effect in the between level was 0.21 that the 95% confidence interval was [0.089, 0.327], exclusive zero; the indirect effect in the within level was 0.11 and that in the 95% confidence interval was [0.047, 0.164], exclusive zero, which indicated the mediation effect of workload was significant in both the between and within levels. Therefore, Hypothesis 2 was supported. In addition, the results demonstrated that leader identification moderated the relationship between authoritarian leadership and nurses’ workload (Model 3: *γ* = 0.25, *p* < 0.01). Therefore, leader identification moderated the effects of authoritarian leadership and workload, and Hypothesis 3 was supported.

**Fig. 2:**
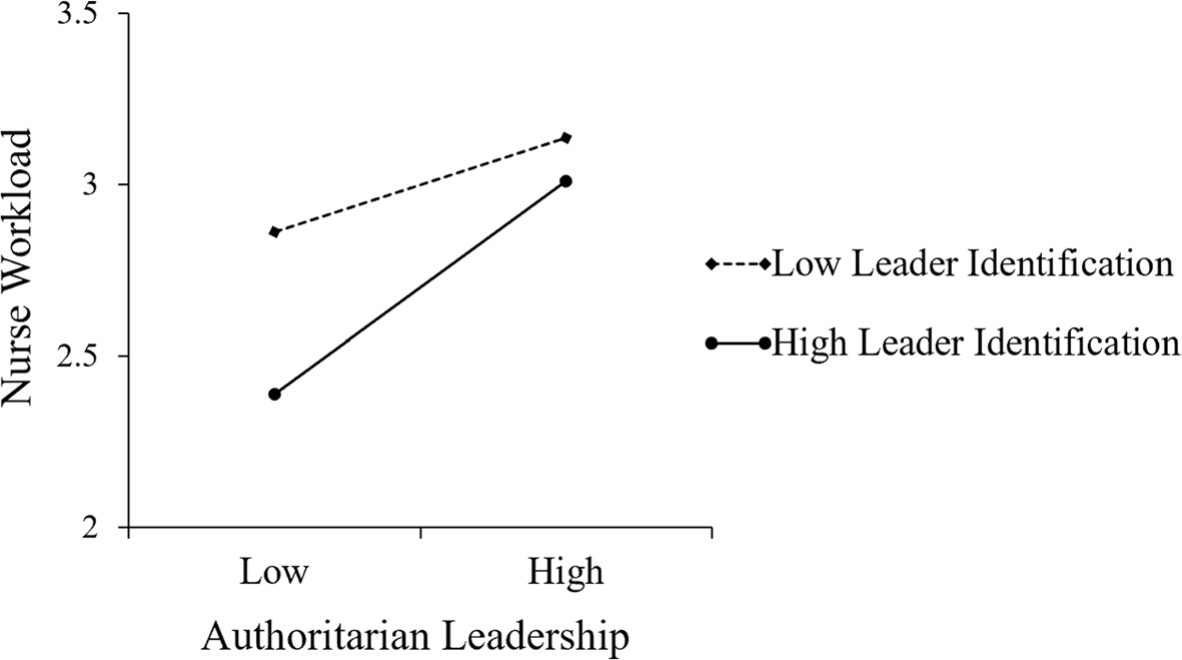
Moderation effect of leader identification between authoritarian leadership and nurses’ workload

The diagram of the moderating effect was plotted to intuitively present the role of leader identification in the relationship between authoritarian leadership and nurses’ workload (see Fig. 2). In the present study, leader identification was divided into high (*M* + 1 *SD*) and low groups (*M*—1 *SD*), and a simple slope test was conducted. The results indicated a positive impact of authoritarian leadership on workload, regardless of whether the subject belonged to the low (*γ* = 0.18, *p* < 0.05) or high (*γ* = 0.41, *p* < 0.01) groups of leader identification. With an increase in leader identification, the effect of authoritarian leadership on workload gradually increased.

## Discussion

### General discussion

The high incidence of presenteeism has been widely proven among nurses. Although the positive effects of presenteeism on performance evaluation have been examined in recent research [58], providing healthcare services under poor health may interfere with the work efficiency of healthcare professionals, with the consequent risk to patients and impairment of the quality of healthcare delivery [10, 59]. On the contrary, sickness absence may sometimes be an effective way to recuperate from poor health and regain high work efficiency, although it would challenge the daily management of human resources and its implementation would be subject to various factors [60]. The present study emphasized nurses’ presenteeism based on the Chinese workplace culture and examined the relationship mechanism between authoritarian leadership and nurse presenteeism. The results suggest that authoritarian leadership has a significant positive correlation with nurse presenteeism, that authoritarian leadership could indirectly increase presenteeism via aggravating subordinates’ workload, and that leader identification played a moderating effect between authoritarian leadership and workload. The present study is expected to contribute to scientifically preventing and managing nurse presenteeism to improve the quality of nursing service.

First, the present study indicated that presenteeism varied significantly according to participants’ demographic characteristics, such as marital status, educational level, technological title, and general health. The results illustrate that presenteeism frequently occurs in married nurses rather than in unmarried nurses, which is consistent with previous studies. This may be attributed to the fact that married nurses are more likely to face work-family conflicts, as they take on multiple roles and functions, such as taking responsibility for the partner or parents, which may create motivation and pressure to work hard [40, 61]. In addition, nurses with higher educational levels and technological titles exhibit more presenteeism behaviors, which could be explained by their irreplaceability for certain tasks. These nurses tended to be attached to responsibilities that are difficult for others to fulfil; they were also highly controlled by their work tasks and felt greater time pressure, thus persisting to work even in ill health [62]. The negative relationship between health status and presenteeism was proven to be consistent with the results of this investigation [63]. Even from the perspective of the connotation of presenteeism, presenteeism has to occur when individuals have health problems. Therefore, health status is the most important prerequisite of presenteeism. In addition, the present study also found that presenteeism was positively correlated with age and tenure. When viewed through the lens of the Chinese workplace culture, older and longer-tenured nurses have a higher acceptance of the effort-related work values and collectivism that are advocated in the organization. Along with activating the work motivation of individuals and enhancing work outcomes for their organizations, these values also contribute to the long-hour working culture that promotes individuals to work under unhealthy conditions. Meanwhile, the older and longer-tenure nurses seemed to form a relatively negative moral perspective of work absences, thus participating in presenteeism to avoid absenteeism [64]. Another cause could be their sense of fear that frequent absenteeism might cause them to lose their jobs; thus, they tended to force themselves to work even in ill health [61, 65].

Second, the results confirmed the positive relationship between authoritarian leadership and nurses’ presenteeism and the mediating effect of workload in the relationship between authoritarian leadership and presenteeism, thus supporting Hypotheses 1 and 2. According to the conservation of resources theory [37], tension and stress responses could be caused by a lack of recourse and threatened by the resources; thus, nurses may have been motivated to avoid further loss of resources. Authoritarian leaders have very few internal constraints but an underlying need to control; further, they allocate resources to subordinates, often through personal decision [42]. Therefore, such a leadership style may aggravate the threat of resource loss for subordinates, which could lead them to insist on working even when ill to cover the shortage of resources. Similarly, the theory of paternalistic leadership demonstrated that strong authoritarian leadership invariably manifests in behaviors such as ignoring subordinate’s suggestions, belittling their dedication, and insisting on absolute obedience, which is an antecedent factor of abusive supervision [21]. In accordance with the theory of resource conservation, authoritarian leadership is easily considered a stressor for subordinates owing to the strong control and high pressure they experience. The strict requirements regarding work performance of authoritarian leaders bring tension and a stressed perception of work requirements to subordinates. As a consequence, heavy workload would be perceived by subordinates with authoritarian leaders, and the presenteeism of subordinates would be indirectly increased due to an increase in workload. These observations may provide valuable insights for healthcare-related specialists and policymakers involved in training and selecting leaders in nursing, as excessive authoritarianism and centralization play an adverse role in healthy working behavior and the development of a healthy working environment.

Third, the present study found that leader identification played a moderating role between authoritarian leadership and nurses’ presenteeism, thus supporting Hypothesis 3. It is noteworthy that leadership effectiveness has been reinforced by leader identification in previous studies [46, 66]. However, the role of leader identification is not always positive and protective, and the relationship between dark leadership and its outcomes could also be heightened from the observations in this research. This is because high leader identification avail to play the model role of leaders [67], regardless of whether the outcomes of the leadership are dark or bright. When subordinates under authoritarian leadership are faced with high leader identification, they prefer to experience the heavier workload that authoritarian leaders expect. Therefore, the impact of leaders on subordinate nurses’ working behavior needs to be further explored, and an effective management system is required to ensure nursing quality and nurse health.

### Theoretical implications

The findings of this study have both theoretical and practical implications. Although research on presenteeism has been conducted recently, scholars from multiple fields have attempted to form a comprehensive and thorough understanding of presenteeism. In this study, we emphasized the impact of authoritarian leadership on presenteeism, thereby enriching the literature on presenteeism and authoritarian leadership in the following ways. First, previous studies were mainly conducted at the individual level [68, 69]. Despite studies noticing the influence of team-related factors (such as leader behaviors and team climates) on presenteeism, only a handful of studies have been conducted using a cross-level design [12, 70]. This study clarified the cross-level impact of authoritarian leadership on presenteeism, thereby elucidating the antecedents of presenteeism and enriching the outcomes of authoritarian leadership. Second, the origin of authoritarian leadership and prevalence of presenteeism are inseparable from the Chinese workplace culture. In this study, an integrated model that combines a typical leadership and presenteeism was formulated to explain the occurrence of presenteeism from a novel perspective. In this research framework, the mediation effect of workload and moderation effect of leader identification were also considered to explain the relationship between authoritarian leadership and presenteeism. Meanwhile, the present study’s findings will not only enrich empirical research on presenteeism but also that on authoritarian leadership in occupational health psychology and other related areas.

### Practical implications

This study has three main practical implications. First, the research variables were selected from participants’ cultural context, which contributes to preventing and managing nurses’ presenteeism from a cultural perspective. Thus, we suggest that managers in the health and medicine fields should be made aware of the occurrence mechanism of presenteeism in their specific workplace cultures, particularly in a society that attaches great importance to hard work and working overtime [61]. Second, the participants in this study were nurses, who are some of the most critical actors in universal healthcare; however, presenteeism is frequent in this profession [4, 71]. Keeping a watchful eye on presenteeism in the nursing field could enhance the quality of nursing care and contribute to the implementation of the “Healthy China Initiative.” Finally, this research analyzed the factors influencing presenteeism and further inspected the impact mechanism of authoritarian leadership on presenteeism. These observations indicate that excessive control of subordinates could promote the incidence of presenteeism and impede the progress of healthcare services. Hence, providing more valuable resources for nurses and enforcing a flexible management system are needed to decrease the prevalence of presenteeism.

### Limitations and future research

Although this study enriches relevant research on authoritarian leadership and presenteeism, its potential limitations should be considered. The first is the universality of the sample. Although the participants were recruited from central China, which is often regarded as the epitome of China in multiple aspects [72], they were all recruited from the same province; thus, the sample size and the region of participants should be expanded in future research. Next, the present study examined the influencing mechanism of authoritarian leadership on nurses’ presenteeism; however, it is only one type of leadership, thereby making the findings inadequate to establish a comprehensive framework of the impact of leadership on nurses’ presenteeism. In addition, the importance of social interactions for presenteeism has been verified by recent studies, which began paying attention to the impact of colleagues’ factors on presenteeism [73]. In future research, more leadership styles and behaviors should be examined, along with what role social interactions play in nurses’ presenteeism, by building an integrated model comprising the factors of leaders, colleagues, and employees. Meanwhile, the cultural foundation underlying the occurrence of presenteeism should be explored further. Besides, although the cross-level role of leaders in presenteeism was preliminarily verified among nurses, this study collected information from one source. It is noteworthy to explore the relationship between presenteeism and behavioral congruence exhibited by the leader vis-à-vis what subordinates perceived.

## Conclusion

Existing studies have confirmed the serious adverse effects of nurses’ presenteeism on individual health, patient safety, and organizations. This study examined the antecedents and mechanisms of nurses’ presenteeism from the perspective of workplace culture. The results demonstrated that authoritarian leadership directly increased the incidence of presenteeism among nurses. Simultaneously, authoritarian leadership contributed to the workload of nurses and indirectly increased the occurrence of presenteeism. Leader identification moderated the relationship between authoritarian leadership and nurses’ workload. Our findings suggest that leaders excessively control resources, which has the disadvantage of shaping their subordinates’ healthy working behaviors.

## Data Availability

The datasets generated for this study are available on request to the corresponding author.
