# Correction: Authoritarian leadership and nurse presenteeism: the role of workload and leader identification

**DOI:** 10.1186/s12912-022-01166-9

**Published:** 2023-01-04

**Authors:** Geyan Shan, Wei Wang, Shengnan Wang, Yongjun Zhang, Shujie Guo, Yongxin Li

**Affiliations:** 1 Business School, Henan University, Kaifeng, China; 2 Institute of Psychology and Behavior, Henan University, Kaifeng, China; 3 Institute of International Education, Henan University of Animal Husbandry and Economy, Zhengzhou, China; 4 Department of Outpatient, Henan Provincial People’s Hospital, People’s Hospital of Zhengzhou University, Zhengzhou, China

**Correction: BMC Nurs 21, 337 (2022)**

**https://doi.org/10.1186/s12912-022-01119-2**

Following publication of the original article [1], the authors reported that the order of the 3rd affiliation and the 4th affiliation should be exchanged. The correct author affiliations have been provided in this Correction.

The original article [1] has been corrected.
